# Intracellular Properties of Deep-Layer Pyramidal Neurons in Frontal Eye Field of Macaque Monkeys

**DOI:** 10.3389/fnsyn.2021.725880

**Published:** 2021-09-21

**Authors:** Charlotte Piette, Marie Vandecasteele, Clémentine Bosch-Bouju, Valérie Goubard, Vincent Paillé, Yihui Cui, Alexandre Mendes, Sylvie Perez, Silvana Valtcheva, Hao Xu, Pierre Pouget, Laurent Venance

**Affiliations:** ^1^Dynamics and Pathophysiology of Neuronal Networks Team, Center for Interdisciplinary Research in Biology (CIRB), College de France, CNRS, INSERM, PSL University, Paris, France; ^2^INSERM, CNRS, Institut du Cerveau, Sorbonne Université, Paris, France

**Keywords:** frontal eye field, primate, pyramidal cells, visual cortex, neuron classification, electrophysiology, intrinsic membrane properties

## Abstract

Although many details remain unknown, several positive statements can be made about the laminar distribution of primate frontal eye field (FEF) neurons with different physiological properties. Most certainly, pyramidal neurons in the deep layer of FEF that project to the brainstem carry movement and fixation signals but clear evidence also support that at least some deep-layer pyramidal neurons projecting to the superior colliculus carry visual responses. Thus, deep-layer neurons in FEF are functionally heterogeneous. Despite the useful functional distinctions between neuronal responses *in vivo*, the underlying existence of distinct cell types remain uncertain, mostly due to methodological limitations of extracellular recordings in awake behaving primates. To substantiate the functionally defined cell types encountered in the deep layer of FEF, we measured the biophysical properties of pyramidal neurons recorded intracellularly in brain slices issued from macaque monkey biopsies. Here, we found that biophysical properties recorded *in vitro* permit us to distinguish two main subtypes of regular-spiking neurons, with, respectively, low-resistance and low excitability vs. high-resistance and strong excitability. These results provide useful constraints for cognitive models of visual attention and saccade production by indicating that at least two distinct populations of deep-layer neurons exist.

## Introduction

It is becoming increasingly important to determine the identity of cortical neurons involved in a wide range of brain functions. The neocortex is comprised of different classes of pyramidal cells and interneurons, and distinguishing between these groups of neurons in recordings made from awake, behaving animals is a key issue. Neocortical neurons have been distinguished by their firing patterns and morphology (Connors and Gutnick, 1990; Krimer et al., 2005; Chang and Luebke, 2007; Zaitsev et al., 2012), laminar distribution (Dow, 1974; Bullier and Henry, 1979; Condé et al., 1994), molecular composition (Cauli et al., 1997; Martina et al., 1998), functional property (González-Burgos et al., 2005), as well as developmental origin (Letinic et al., 2002). In the oculomotor field of research, it has been demonstrated that several cortical areas and subcortical regions contribute to the visual-motor mapping. One such area, the frontal eye field (FEF), contains at least three main functional types of neurons: visual, movement, and visuo-movement neurons (Goldberg and Bushnell, 1981; Bruce and Goldberg, 1985; Segraves and Goldberg, 1987; Schall, 1991; Segraves, 1992; Kodaka et al., 1997; Umeno and Goldberg, 1997; Hanes et al., 1998; DiCarlo and Maunsell, 2005). The visual and visuo-movement neurons select the target of search by increasing their firing rate in response to the presence of the target in their receptive fields (RFs) relative to when a distractor is located in their RFs (e.g., Schall and Hanes, 1993; Thompson et al., 1996). A different population of neurons, called movement neurons, increases their firing rate leading up to saccades into their movement fields (MFs) (e.g., Hanes and Schall, 1996). Finally, visuo-movement neurons also increase their firing rate leading up to saccades while they also respond to the presence of the target in RFs (Everling and Munoz, 2000; Sato et al., 2001; McPeek, 2006; Ray et al., 2009).

Some recent works have also shown that visuo-movement neurons tend to have the thinnest spikes, consistent with a role in local processing while movement neurons were found to have the widest spikes, consistent with their role in sending eye movement commands to subcortical structures such as the superior colliculus. Finally, visual neurons had wider spikes than visuo-movement neurons, consistent with their role in receiving projections from the occipital and parietal cortex (Cohen et al., 2009). These distinctions between these cell types have relied largely on firing-rate patterns or spike waveforms indirect analysis as opposed to inherent biophysical properties of the neurons being studied. As a consequence, as in the primary motor cortex where the report of thin-spiked pyramidal cells urge caution in matching extracellular recording-based and anatomically defined cell types (Lemon et al., 2021), some disagreements persist about the reliability of the distinction between FEF neuron types solely based on functional rate pattern responses (Lowe and Schall, 2018). To substantiate the functionally defined cell types encountered in FEF, we measured intracellular properties of FEF neurons recorded *in vitro* using whole-cell patch-clamp recordings in FEF acute brain slices issued from macaque monkey biopsies. The relationship between intracellular properties and functional properties of FEF is a critical missing piece of information to construct a valid physiological model of visual target selection and saccade programming.

## Materials and Methods

### Ethics Statement

All experiments were carried out in accordance with the recommendations contained in the European Community Council Directives of 1986 (86/609/EEC) and the NIH Guide for the Care and Use of Laboratory Animals and were approved by the French Animal Ethics Committee of INSERM. The animals were housed under conditions of constant temperature (21 ± 1°C), humidity (55 ± 5%), and air replacement (16 times/h), on a 12-h light/12-h dark cycle with access *ad libitum* to food and water.

### Animals

Biopsies were obtained at the time of their euthanasia from 8 long-tailed macaque monkeys (*Macaca fascicularis*). All animals were involved in tracer and/or lesion studies in ethically approved projects, 6 animals were 5–6 years old and their weights ranged from 3 to 5 kg. Our work benefits from these studies to obtain biopsies at the time of the terminal experiments of other studies.

### Surgery and Brain Slice Preparation

Following injections of ketamine hydrochloride (25 mg/kg), atropine sulfate (0.05 mg/kg), an endotracheal tube was inserted, and the animal was placed in a stereotaxic frame. Anesthesia was maintained with 2% isoflurane in 30% O_2_/air. A large craniotomy was performed over the prefrontal cortex, and a small block of tissue containing both lateral banks of arcuate sulcus (areas 8 and 46) as well as part of area 9 (Walker 1940) was carefully excised. The tissue block was placed in a 95% CO_2_/5% O_2_-bubbled, ice-cold solution consisting of (in mM) 125 NaCl, 2.5 KCl, 25 glucose, 25 NaHCO_3_, 1.25 NaH_2_PO_4_, 2 CaCl_2_, 1 MgCl_2_, 1 pyruvic acid.

After the craniotomy, the animal was given an overdose of pentobarbital (30 mg/kg) and was perfused transcardially with ice-cold–modified artificial cerebrospinal fluid. A tissue block containing the portions of areas 9 and 46 non-homotopic to the first biopsy was quickly excised. Sagittal slices (330 μm) were cut using a vibrating blade microtome (VT1200S, Leica Microsystems, Nussloch, Germany). Brains were sliced in a 95% CO_2_/5% O_2_-bubbled, ice-cold cutting solution containing (in mM) 125 NaCl, 2.5 KCl, 25 glucose, 25 NaHCO_3_, 1.25 NaH_2_PO_4_, 2 CaCl_2_, 1 MgCl_2_, 1 pyruvic acid, and then transferred into the same solution at 34°C for 1 h and then kept at room temperature.

### Electrophysiological Recordings

Whole-cell patch-clamp recordings were performed as previously described (Paillé et al., 2013), using borosilicate glass pipettes of 4–6 MΩ resistance, filled with (in mM): 105 K-gluconate, 30 KCl, 10 HEPES, 10 phosphocreatine, 4 Mg-ATP, 0.3 Na-GTP, 0.3 EGTA (adjusted to pH 7.35 with KOH), and 0.5% biocytin. The composition of the extracellular solution was (mM): 125 NaCl, 2.5 KCl, 25 glucose, 25 NaHCO_3_, 1.25 NaH_2_PO_4_, 2 CaCl_2_, 1 MgCl_2_, 10 μM pyruvic acid bubbled with 95% O_2_ and 5% CO_2_. Signals were amplified using EPC9-2 and EPC10-4 amplifiers (HEKA Elektronik, Lambrecht, Germany). All recordings were performed at 34°C, using a temperature control system (Bath-controller V, Luigs and Neumann, Ratingen, Germany) and slices were continuously superfused with extracellular solution, at a rate of 2–3 ml.min^–1^. Slices were visualized under an Olympus BX51WI microscope (Olympus, Rungis, France), with a 4 × /0.13 objective for the placement of the stimulating electrode and a 40 × /0.80 water-immersion objective for the localization of cells for whole-cell recordings. Current–clamp recordings were sampled at 2.5 kHz and voltage–clamp recordings were sampled at 10 kHz, with the Patchmaster v2 × 32 program (HEKA Elektronik). Glutamate transmission blockers 6-Cyano-7-nitroquinoxaline-2,3-dione (CNQX, 10 μM, Tocris, Ellisville, MO, United States) and DL-2-amino-5-phosphono-pentanoic acid (D-AP5, 50 μM, Tocris) were bath-applied and responses were measured after 5 min of application.

### Electrophysiological Data Analysis

Off-line analysis was performed using Patchmaster (Heka Elektronik), Igor-Pro 6.0.3 (Wavemetrics, Lake Oswego, OR, United States) and MATLAB (The Mathworks).

Pyramidal neurons were identified in slices by their morphology and basic electrophysiological characteristics to distinguish them from interneurons (Cohen et al., 2009; Mueller et al., 2020): patched cells were pre-selected by their large soma with triangular shape through a visual inspection using infrared DIC video-microscopy. Offline analysis of AP features was then used to exclude interneurons, namely if the spike half-width was < 0.7 ms and/or the rise/decay slope ratio was < 1 in the absence of an ADP. Only cells that had a resting membrane potential (RMP) of less than −50 mV (unless spontaneously active), and an AP overshoot were included. 50 cells were retained for clustering analysis but not all cells were held for sufficient time to allow all protocols to be completed (Supplementary Table 1).

#### Passive and Active Membrane Properties

**FIGURE 1:**
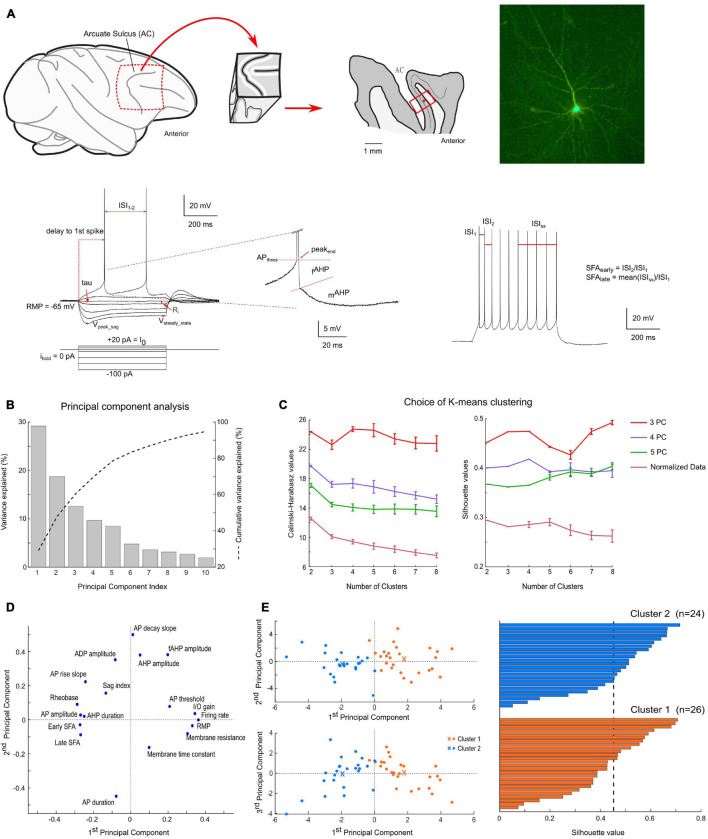
Methodology and results of the clustering analysis. **(A)** Brain slice preparation; from left to right: sagittal view of the frontal cortex of macaque monkey (circle highlights the FEF localization, from which a biopsy is extracted), sagittal section through the FEF biopsy (layer 4 is indicated by a thin black line, the recording location is indicated by a red dot) and example of a recorded pyramidal neuron after fluorescent revelation of biocytin filling; Bottom: electrophysiological response of a pyramidal neuron to current step injections (left: up to rheobase; right: first train of at least 9 spikes; center: close-up on a spike AHP). Parameters used for analysis (in red) are further described in section “Materials and Methods.” **(B)** Percentage of variance explained by each principal component of the principal component analysis. *k*-means clustering was performed on up to the first five principal components, which explained more than 80% of the total variance. **(C)** Variation of the Calinski-Harabasz index and average silhouette values as a function of the number of clusters and PCA results. These two indices were used to define the optimal number of clusters in the data. **(D)** Projection of the 18 electrophysiological parameters used for clustering on the first two principal component axes. **(E)** Results of the *k*-means clustering algorithm applied to the first three principal components with 2 clusters (*n* = 26 cells for cluster 1, in orange; and *n* = 24 for cluster 2, in blue). As visible on the projection plane (left), the first principal component contributes the most to the discrimination between the two clusters. The centroids of the clusters are indicated by orange and blue crosses. The silhouette scores (right) indicate a good level of compactness of each cluster.

Cells were recorded in a current-clamp mode for their electrophysiological characterization. Resting membrane potential (RMP) was determined by measuring the membrane voltage in the absence of current input. For the PCA and clustering analysis, the membrane potential of spontaneously active cells was measured by excluding the spiking periods, but is not considered for the RMP comparison between clusters. A series of 500-ms hyperpolarizing and depolarizing current steps ranging from −100 in 10 pA increments were applied to the cell. For spontaneously active cells, steps were applied on top of a holding current to maintain them at rest (−60/−70 mV). Input resistance (Ri) was calculated from a single sweep as the ratio of the steady-state membrane voltage response to the current applied after injecting a small hyperpolarizing current (10–20 pA). Membrane time constant (tau) was determined on the same step by fitting the membrane potential response to a single-exponential function. Sag index was measured on a 100 pA hyperpolarizing current step and expressed as the percent of the peak voltage response that is repolarized at steady state: (V_peak sag_ − V_steady state_)/(V_peak_ − V_baseline_)^∗^100. The rebound index was measured at the offset of the same step as the maximal positive voltage response above baseline (V_peak_rebound_) normalized by the amplitude of the steady-state response: (V_peak_rebound_ − V_baseline_)/(V_peak_ − V_baseline_)^∗^100. The rheobase (I_0_) was the minimal current step that evoked firing (for spontaneously active cells, it was corrected by the holding current for the PCA and clustering analysis, thus yielding negative rheobase values; for the comparison between the two clusters, rheobase was equal to 0 pA for spontaneously active cells). The delay to the first spike (from the step onset to the first spike) was measured at I_0_, as well as AP properties. Action potential threshold (AP_thres_) was chosen as the membrane potential at which the rate of voltage rise (dV/dt) reached 10 mV/ms. Action potential amplitude (AP_amp_), rise time, and rise slope were measured from the AP_thres_ to the peak of the AP. The AP decay time and decay slope were measured from the AP_peak_ to the interpolated point where the AP decay crosses AP_thres_ (peak_end_). Action potential duration (AP_dur_) was measured as the spike width at its half-amplitude. Amplitude and duration of the afterhyperpolarization (AHP_amp_ and AHP_dur_) were measured from the peak_end_ to the through after the spike. We distinguished 1 to 3 components in the AHP: a fast component of the AHP (fAHP) present in all cells, a slower medium component (mAHP) in most cells, and an afterdepolarization (ADP) between the fAHP and the mAHP in some cells. The fAHP amplitude was measured between peak_end_ and either the trough of the AHP for single-component AHPs, the onset of the mAHP (marked slowing in the voltage drop) for 2-component AHPs, or the onset of the ADP for 3-components AHPs. The ADP amplitude (when present) was measured between the end of the fAHP and the peak of the ADP. The mAHP amplitude was measured between either the end of the fAHP (2-component AHPs) or the peak of the ADP (3-component AHP), and the next trough of the AHP (see Figure 1). The rectification index was calculated as the ratio of the IV curve slope between −20 and 0 pA injected current over the IV curve slope between −100 and −80 pA injected current.

#### Subthreshold Frequency-Response Curves

We used the impedance amplitude profile (ZAP) method to characterize the resonant behavior of pyramidal cells (Puil et al., 1988; Hutcheon and Yarom, 2000). A sinewave current of fixed amplitude and 30-s duration, with a linear increase of the frequencies ranging from 0.1 to 50 Hz was used. A single zap voltage response was analyzed for each neuron. The impedance profile Z(f) was calculated from the ratio of the Fourier transforms of the voltage response and zap current; its absolute value is the impedance magnitude and its imaginary part corresponds to the phase shift between the input current and voltage response. Plotted against each other, the two components form the “impedance locus diagram”. Peak in the impedance power, indicative of membrane resonance, was detected and used to define the resonant frequency (Hutcheon and Yarom, 2000). The Q factor, a measure of resonance strength, was calculated as the ratio of the impedance magnitude at the resonant frequency over the impedance magnitude at 0.5 Hz. For a more precise determination of Q, the impedance profile of each neuron was fitted with a polynomial curve (degree 8) between 0.5 and 20 Hz and the peak value was calculated. A cutoff criterion of Q ≥ 1.1 was used to differentiate resonant from non-resonant cells (Vera et al., 2014). Frequencies below 0.5 Hz were not plotted in the impedance and phase profiles graphs to avoid low frequency distortions.

#### Firing Pattern Properties

The first interspike interval (ISI_1__–__2_) was measured between the first and second spikes at the minimal suprathreshold current that elicited at least 2 spikes. The spike frequency adaptation indices were determined at the minimal depolarizing current step that elicited at least 9 spikes: the early spike frequency adaptation (SFA_early_) was calculated as the ratio of the second ISI to the first ISI, and the late SFA (SFA_late_) as the ratio of the mean of ISIs during the last half of the pulse (ISI_ss_) to the first ISI. The firing rate at + 40 pA was calculated using the number of action potentials elicited during the 500 ms pulse (mean firing frequency), with a current injection of + 40 pA from rheobase. The f-I curve was constructed by plotting the mean firing frequency as a function of injected current. The I-O gain corresponds to the slope of the linear fit of the f-I curve, considering the first six current steps after rheobase. If there existed at least one ISI smaller than half of the mean ISI, visible at the start of the spike train, then the cell was included in the proportion of neurons displaying an initial spike doublet. For each current step, the spike frequency adaptation was measured using two different methods: as the ratio of the mean of the three last instantaneous frequencies divided by the first instantaneous frequency (similar to SFA_late_ above), or as the mean of the differences between consecutive ISIs (to minimize the influence of an initial spike doublet, if present). The spontaneous activity characteristics (mean and CV) were calculated on 30-s recording in the absence of holding current.

#### Responses to L2/3 Cortical Stimulation

Voltage-clamp and current-clamp responses to single L2/3 cortical stimulation were first linearly interpolated to reach a time resolution of 0.01 ms. Manually defined cursors were used to detect the onset and peak of the neuron response. When the response showed multiple peaks, the largest one defined the peak of the response. The latency of the response was defined as the interval between the timing of the largest stimulation artifact and the response onset. Extracted parameters consisted of the 20–80% rise slope (obtained from a linear fit, not defined if the response showed multiple peaks in the rising phase), half-duration, area and decay time constant (obtained from a single exponential fit, only fits of trial responses with an r-square > 0.6 were kept). Similar analyses were performed on normalized responses (normalized by the peak amplitude).

EPSPs or spiking events were recorded in response to increased stimulation currents (at least 10 trials for each stimulation current). Whenever possible, we considered the average amplitude of EPSPs eliciting a 0.7–0.9 probability of spiking and the average amplitude of EPSPs at the last stimulation current in which no spiking event was elicited to define an EPSP-spike coupling ratio, equal to the ratio of these latter two average EPSP amplitudes.

Amplitudes of paired-pulse responses in voltage-clamp were measured similarly. To characterize the short-term plasticity properties in the response train, we calculated for each interval (25, 50, 100, or 250 ms), the ratio of the amplitudes of the 2nd–10th EPSC of the train relative to the amplitude of the first EPSC.

### Clustering Analysis

Clustering algorithms were used on standardized data (centered and reduced) from 50 neurons using 18 electrophysiological parameters. The parameters were: (1) RMP, (2) Membrane resistance, (3) Membrane time constant, (4) Rheobase, (5) Sag index, (6) AP threshold, (7) AP amplitude, (8) AP duration at half-width, (9) AP rise slope, (10) AP decay slope, (11) AHP amplitude, (12) fast AHP amplitude, (13) AHP amplitude, (14) AHP duration, (15) Firing rate at + 40 pA from rheobase, (16) SFA_early_, (17) SFA_late_, (18) I/O gain.

The average absolute correlation coefficient between these 19 parameters was 0.30, with only five pairs of parameters for which the absolute value of the correlation coefficient was superior to 0.7. To reduce the dimensionality of the dataset and remove correlations between these parameters, a principal component analysis (PCA) was performed. The first three principal components, explaining 60.5% of the variance, were retained for classification by cluster analysis (Figure 1B). The projection of the 18 electrophysiological parameters onto the first two principal components is indicated in Figure 1D.

Clustering analysis was implemented using the statistics toolbox of Matlab using the k-means algorithm, based on the squared Euclidean distance. We computed the Calinski-Harabasz index, which corresponds to the normalized ratio between the overall between-cluster variance and the overall within-cluster variance. Silhouette scores, a measure of similarity of a sample to points of its cluster when compared to points in other clusters, were measured using the squared Euclidean distance. High average silhouette scores, close to 1, indicate that the clusters are compact and distinct from each other.

Additional tests of the robustness of the clustering results were performed, by varying the number of principal components used or by directly applying the clustering algorithms on the normalized dataset. A comparison of the number of mismatches, average silhouette values and Calinski-Harabasz index is presented in Tables 1, 2. Overall, the clustering results were highly consistent, with only one mismatch. Furthermore, the number of clusters was deduced from the Calinski-Harabasz index and silhouette scores (Figure 1C): in most cases, the optimal number of clusters was 2. Yet, a total of 4 clusters was also found to be an optimal solution when computing these indices on the first three principal components dataset.

**TABLE 1:** Comparison between different clustering algorithms.

Mismatch Counts (2 clusters)	K-3PC	K-4PC	K-5PC	K-Norm
K-3PC	0			
K-4PC	1	0		
K-5PC	1	0	0	
K-Norm	1	0	0	0

**Mismatch Counts (4 clusters)**	**K-3PC**	**K-4PC**	**K-5PC**	**K-Norm**

K-3PC	0			
K-4PC	15	0		
K-5PC	2	15	0	
K-Norm	3	16	1	0

**TABLE 2:** Comparison of clustering quality.

2 clusters	Average silhouette	Scrambled silhouette (± SD)	Calinski-Harabasz index	Scrambled Calinski-Harabasz index (± SD)
K-3PC	0.45 (*p* = 0.13)	0.39 ± 0.05	24.4 ± 0.05 (*p* = 0)	16.2 ± 1.5
K-4PC	0.40 (*p* = 0.06)	0.33 ± 0.05	19.7 ± 0.1 (*p* = 0)	12.0 ± 1.2
K-5PC	0.37 (*p* = 0.06)	0.29 ± 0.05	17.1 ± 0.3 (*p* = 0)	9.7 ± 0.9
K-Norm	0.29 (*p* = 0.03)	0.15 ± 0.05	12.5 ± 0.2 (*p* = 0)	4.3 ± 0.4

**4 clusters**	**Average silhouette**	**Scrambled silhouette (± SD)**	**Calinski-Harabasz index**	**Scrambled Calinski-Harabasz index (± SD)**

K-3PC	0.47 (*p* = 0.27)	0.44 ± 0.04	24.7 ± 0.3 (*p* = 0.02)	19.6 ± 2.5
K-4PC	0.42 (*p* = 0.01)	0.36 ± 0.03	17.3 ± 0.6 (*p* = 0)	12.6 ± 1.1
K-5PC	0.37 (*p* = 0.03)	0.31 ± 0.03	14.1 ± 0.5 (*p* = 0)	9.7 ± 0.8
K-Norm	0.29 (*p* = 0)	0.13 ± 0.02	9.4 ± 0.3 (*p* = 0)	3.6 ± 0.3

We also verified that the quality of the recordings did not affect the clustering results, by estimating the series resistance (R_*series*_) in 47 out of the 50 cells (Supplementary Figure 1). This parameter did not segregate with cluster identity (21 MΩ *vs*. 16.5 MΩ, *p* = 0.055), even though there was a tendency for cluster 1 neurons to present higher series resistance (Supplementary Figure 1A). If this could partially explain the difference observed in spike amplitude (*p* = 0.0064), one can notice the absence of correlation between the series resistance and the input resistance, and a negative correlation between the series resistance and the rheobase (*p* = 0.0116), thus supporting the conclusions that the differences in rheobase between the two clusters are rather due to differences of input resistance rather than to differences in the quality of the recordings (Supplementary Figure 1B). In addition, the R_series_/R_i_ ratio was below 15% for 75% of selected neurons.

### Statistics

Statistical analysis was performed using Matlab 2019 (The Mathworks) or the R-based Jamovi software (The jamovi project, 2021). In all cases “n” refers to a single cell experiment from a single slice.

When comparing electrophysiological features between the two groups, reported *p*-values were calculated using a non-parametric Mann-Whitney test. Chi-square tests were used to compare the proportions of resonant neurons, or spontaneously active neurons between clusters. A 2-way repeated measures ANOVA was used to compare the I-V curves (injected current X cluster identity). A linear mixed model (LMM) was used to compare the f-I curves (fit by REML, random effect of the neuron identity and fixed effects of injected current X cluster identity tested using omnibus *F*-test with Satterthwaite method for the degrees of freedom). A generalized linear mixed model was used to compare the proportion of bursting cells (logistic model, the random effect of the neuron identity and fixed effects of injected current + cluster identity tested using omnibus chi-square tests; the interaction between factors could not be tested, as the corresponding model did not converge). LMMs were used to compare the adaptation ratio measures (fit by REML, random effect of the neuron identity and fixed effects of the number of spikes in the train × cluster identity tested using omnibus *F*-test with Satterthwaite method for the degrees of freedom). A 3-way repeated measures ANOVA was used to analyze the responses to trains of cortical stimulation (pulse number × stimulation interval × cluster identity), and 2-way repeated measures ANOVAs were further performed to analyze separately the initial and final paired pulse ratio in response to trains of stimulation (stimulation interval × cluster identity).

### Histology

Biocytin (Sigma) 5 mg/ml was dissolved in the pipette solution and cells were filled during at least 20 min of recording. Subsequently, slices were fixed overnight in 2% paraformaldehyde at 4°C. Biocytin-filled cells were visualized using streptavidin-alexa488 (Invitrogen, Carlsbad, CA, United States), incubated for 2 h at room temperature. Slices were mounted on glass slides for examination under an epifluorescence microscope (DMRB, Leica).

## Results

### Electrophysiological Classification of FEF Deep-Layer Pyramidal Neurons Using Cluster Analysis

Pyramidal neurons of the FEF deep layer (*n* = 50) were recorded by whole-cell patch-clamp at 34°C in parasagittal brain slices from tissue block containing the portions of areas 9 and 46 non-homotopic from adult macaque monkeys (*n* = 8 animals). In a subset of experiments (*n* = 5), the pyramidal nature and deep-layer localization of recorded neurons were confirmed by biocytin staining injected through the recording pipette (Figure 1A).

**FIGURE 2:**
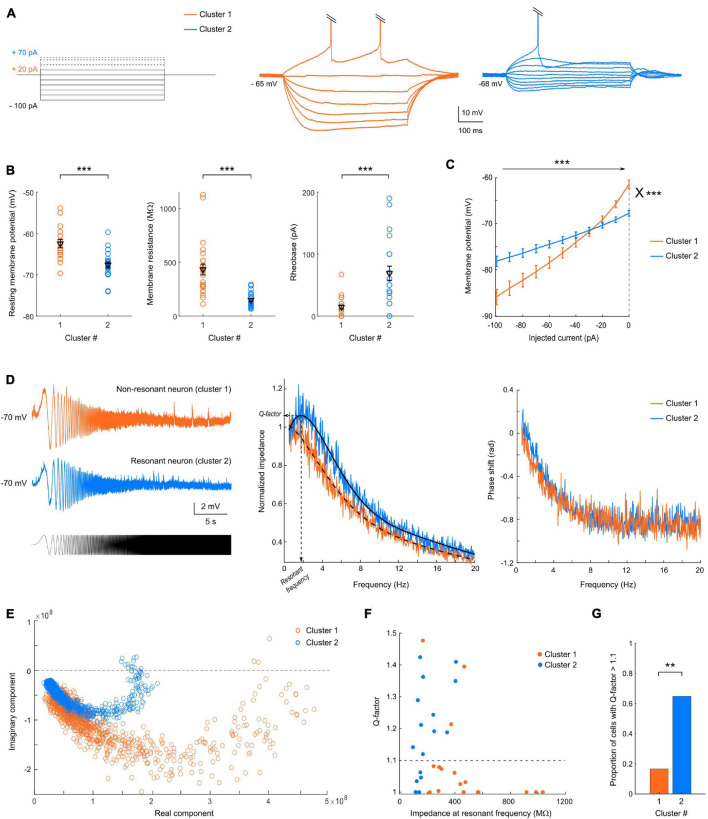
The two clusters exhibit different passive membrane properties. **(A)** Representative responses of cluster 1 (orange, center) or cluster 2 (blue, right) neuron to current step injections (left), from –100 pA to rheobase. **(B)** Scatter plots of the passive membrane properties showing significant differences between the two clusters, with mean and SEM indicated in black. Cluster 1 neurons, indicated in orange, have a more depolarized resting membrane potential (*p* < 0.001), a higher membrane resistance (*p* < 0.0001) and a lower rheobase (*p* < 0.0001) than cluster 2 neurons, shown in blue. **(C)** Average IV-curves show an inward rectification for cluster 1, but a linear relationship between the injected current and membrane potential for cluster 2, and highlight the differences in membrane resistance and resting membrane potential. **(D)** (left) Example of voltage responses to a sinusoidal chirp current injection (bottom) for a representative non-resonant neuron belonging to cluster 1 and a resonant neuron from cluster 2. (Center) Average resonant impedance profiles for the two clusters (black: smoothing average); the resonant frequency and Q-factor are indicated for cluster 2. (right) Phase shift of the voltage waves relative to the injected current as a function of frequency. **(E)** Impedance vectors in the complex plane. The distance to the origin corresponds to the impedance magnitude and the angle with the real axis corresponds to the phase shift. Frequency increases in the clockwise direction. **(F)** Distribution of Q-factors as a function of the impedance amplitude at the resonant frequency and of the clustering classification. **(G)** Proportion of resonant cells (Q factors > 1.1), in cluster 1 and 2 (Chi-square test = 8.41; *p* = 0.0037). All data: mean ± SEM. *p < 0.05; **p < 0.01; ***p < 0.001.

**FIGURE 3:**
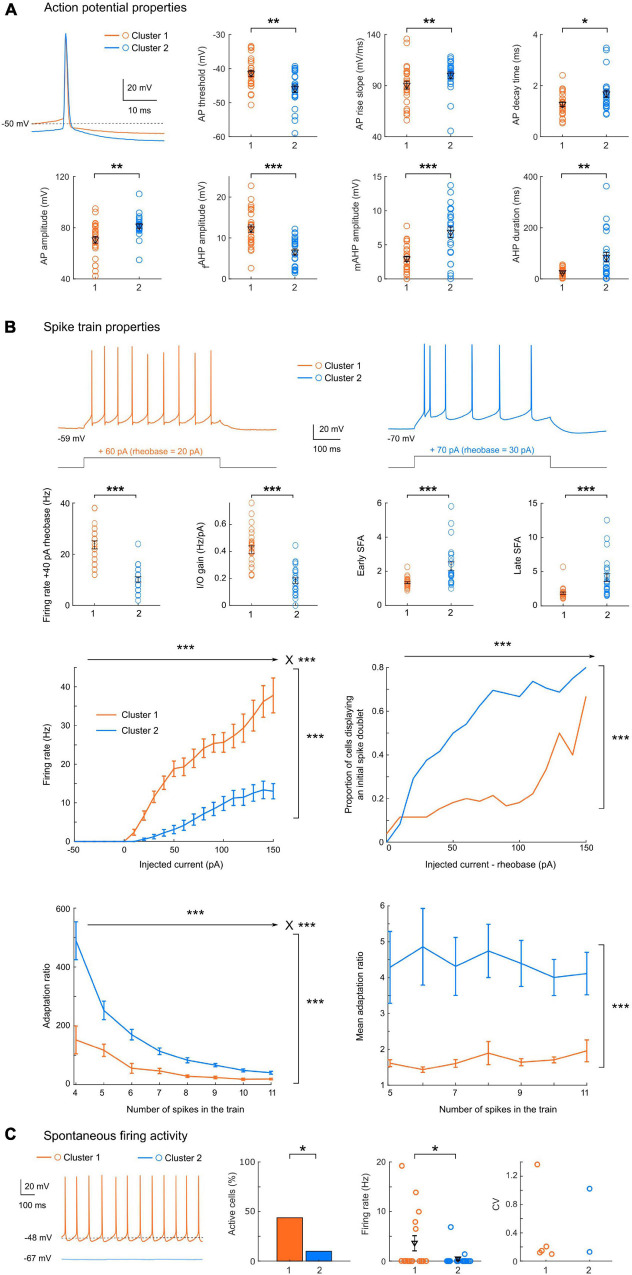
Cluster 1 neurons have a higher excitability with less bursting and firing adaptation. **(A)** Representative action potentials for clusters 1 or 2, and scatter plots of the action potential properties showing significant differences between the two clusters, with mean and SEM indicated in black. Cluster 1 neurons have a more depolarized action potential threshold (*p* = 0.0039), a smaller AP amplitude (*p* = 0.0011), a slower rise slope (*p* = 0.0090) but a shorter decay time (*p* = 0.0237) than cluster 2 neurons. The AHP also presented different characteristics, with a larger fast component but a smaller mAHP in cluster 1 cells (*p* < 0.0001 for both), associated to an overall shorter duration (*p* = 0.0033). **(B)** Spike train properties showing significant differences between the two clusters. Top: Representative spike trains for cluster 1 or 2, in response to a current step injection of + 40 pA above the rheobase, and scatter plots with mean and SEM indicated in black. Cluster 1 neurons display an elevated excitability (with a higher firing rate at + 40 pA from rheobase and a larger I/O gain, *p* < 0.0001 for both). In addition, the spike frequency adaptation for early and late ISIs are significantly smaller in cluster 1 neurons (*p* < 0.0001 for both). Middle: spike train mean firing rate (left, 2-way repeated measures ANOVA: *p* < 0.0001 for main effects of injected current, cluster identity and their interaction) and proportion of cells displaying an initial spike doublet (right, GLMM: *p* < 0.0001 for fixed effect of injected current and cluster identity) as a function of the injected current. Bottom: adaptation ratio measures as a function of the number of spikes in the train (left, LMM: *p* < 0.0001 for fixed effects of number of spikes, cluster identity and their interaction; right, LMM: *p* = 0.001 for fixed effect of cluster identity). **(C)** When spontaneous firing activity was monitored for 30 s (representative examples of a 1 s-epoch from each cluster are shown), the proportion of spontaneously active cells (left) was significantly higher in cluster 1 (*p* = 0.0201), and the average firing rate (center) was significantly higher than in cluster 2 (*p* = 0.0202). Both clusters displayed both regular and irregular firing cells (CV, right). All data: mean ± SEM. *p < 0.05; **p < 0.01; ***p < 0.001.

To characterize the electrophysiological properties of FEF pyramidal cells, we first applied successive hyperpolarizing and depolarizing current steps. Analysis of their responses revealed heterogeneity in both passive and active membrane properties among pyramidal neurons (Figures 1–3). We performed a principal component analysis, using 18 different electrophysiological parameters (see section “Materials and Methods”), for which the average correlation coefficient was 0.3. We then applied a k-means algorithm on the first three principal components, which accounted for 60.5% of the variance (Figure 1B). The optimal number of clusters was defined using two indices: the average silhouette score and the Calinski-Harabasz index, quantifying the compactness of each cluster using, respectively, a distance metric and the variance within and between clusters (Figure 1C). Two clusters of size *n* = 26 and *n* = 24 emerge with strongly significant differences in both their passive and active membrane properties (Table 3 and Figures 1–3). Indeed, we first found significant differences between the two clusters in 12 out of the 18 electrophysiological parameters used for PCA (Table 3). Furthermore, we used this classification to evaluate whether additional electrophysiological properties—not included in the PCA—could further distinguish these two cell types.

**TABLE 3:** Electrophysiological properties.

Mean ± SEM	Cluster 1 (*n* = 26)	Cluster 2 (*n* = 24)	*p*-value (Mann-Whitney)
Resting membrane potential (mV)	−62.5 ±	−67.6 ± 0.7	***p < 0.001
Membrane resistance (MΩ)	432 ± 48	148 ± 14	***p < 0.0001
Membrane time constant (ms)	32 ± 4	24 ± 2	*p* = 0.2600
Sag Index (%)	20 ± 2	22 ± 2	*p* = 0.4203
Rebound Index (%)	35 ± 5	40 ± 6	*p* = 0.4547
Rheobase (pA)	14 ± 3	69 ± 11	***p < 0.0001
Delay to first spike (ms)	114 ± 15	131 ± 12	*p* = 0.1180
AP threshold (mV)	−41.4 ± 0.9	−45.9 ± 1.1	**p = 0.0039
AP amplitude (mV)	70.6 ± 2.6	81.4 ± 1.8	**p = 0.0011
AP duration at half-width (ms)	0.96 ± 0.04	1.10 ± 0.05	*p* = 0.0533
AP rise time (ms)	0.79 ± 0.02	0.82 ± 0.02	*p* = 0.1151
AP rise slope (mV.ms^–1^)	90.5 ± 3.8	100.3 ± 3.2	**p = 0.0090
AP decay time (ms)	1.27 ± 0.09	1.68 ± 0.14	*p = 0.0237
AP decay slope (mV.ms^–1^)	62.4 ± 4.7	54.4 ± 3.5	*p* = 0.3173
AP rise/decay slope ratio	1.61 ± 0.10	1.99 ± 0.12	*p = 0.0275
AHP amplitude (mV)	14.5 ± 0.8	12.5 ± 0.8	*p* = 0.1323
fAHP amplitude (mV)	12.3 ± 0.9	6.6 ± 0.7	***p < 0.0001
ADP amplitude (mV)	0.9 ± 0.2	0.99 ± 0.3	*p* = 0.5574
mAHP amplitude (mV)	2.9 ± 0.4	6.8 ± 0.8	***p < 0.0001
AHP duration (ms)	25 ± 3	85 ± 18	**p = 0.0033
ISI 1–2 (ms)	134 ± 17	150 ± 21	*p* = 0.7122
Firing rate at + 40 pA from rheobase (Hz)	24 ± 2	10 ± 1	***p < 0.0001
Early spike frequency adaptation	1.3 ± 0.1	2.3 ± 0.2	***p < 0.0001
Late spike frequency adaptation	1.9 ± 0.2	4.1 ± 0.6	***p < 0.0001
I/O gain (Hz.pA^–1^)	0.41 ± 0.03	0.18 ± 0.02	***p < 0.0001
Spontaneous frequency (Hz)	5.3 ± 1.7	0.6 ± 0.6	**p = 0.0083

All data: mean ± SEM. *p < 0.05; **p < 0.01; ***p < 0.001.

### Heterogeneity in Electrophysiological Properties

#### Differences in Passive Membrane Properties

Cluster 1 neurons had a significantly more depolarized RMP and higher input resistance (Ri) than cluster 2 cells (−57 ± 2 vs. −66 ± 1 mV, *p* < 0.0001 and 432 ± 48 vs. 148 ± 14 MΩ, *p* < 0.0001, respectively), and consequently a lower rheobase (3 ± 8 vs. 68 ± 12 pA, *p* < 0.0001) (Figure 2A). These results are well summarized in the average I-V curve profiles (Figure 2B), which show a larger slope for cluster 1 [2-way repeated measures ANOVA; main effect of injected current *F*(10, 480) = 268.5, *p* < 0.0001; main effect of cluster identity *F*(1, 48) = 2.55, *p* = 0.1169; interaction between injected current and cluster identity *F*(10, 480) = 41.7, *p* < 0.0001]. Moreover, cluster 1 but not cluster 2 cells displayed an inward rectification (rectification index: 2.2 ± 0.2 vs. 1.3 ± 0.1, *p* < 0.0001). These results suggest that cluster 1 neurons would be more excitable than cluster 2 cells.

#### Subthreshold Resonance Predominates in One Cluster

In a subset of cells (*n* = 35), the impedance amplitude profile was assessed using a chirp protocol (subthreshold current injection of a sinewave of continuously increasing frequency) between 0.1 and 50 Hz during 30 s (Figures 2C,D). The membrane impedance, determined by the cell morphology and voltage-gated ion channels, helps characterizing the gain but also the timing for synaptic integration at a given frequency. We took advantage of this additional protocol (not included in the PCA) to check whether the segregation also stands for impedance characteristics. In non-resonant cells (Figure 2C, example from cluster 1) the voltage response to the chirp stimulus decreases continuously with the stimulus frequency, whereas resonant cells (Figure 2C, example from cluster 2) display a preferred (resonant) frequency indicated by a maximal peak-to-peak voltage response (here ∼2 Hz). Consistently, the average profile of the impedance magnitude normalized by the impedance at 0.5 Hz presents a clear bump for cluster 2 neurons while a monotonic decrease is visible for cluster 1 neurons (Figure 2D). In both groups, the average phase shift increases monotonically with the oscillation frequency until reaching a plateau at 10 Hz (Figure 2D), but cluster 2 neurons showed a slower increase in the phase lag relative to the current, with even slightly positive phase values at the lowest frequencies, which indicate that inductive properties of ionic channels responsible for the resonance dominate over the passive low-pass filtering (Figures 2D,E). The degree of resonance, assessed by the Q-factor, and equal to 1 in absence of resonance, was significantly different between the two clusters (*p* = 0.0203, Figure 2F). Notably, 65% of pyramidal neurons of cluster 2 (*n* = 11 out of 17 cells tested) displayed a resonance (Q-factor > 1.1) at 2.13 ± 0.26 Hz (*n* = 11), while only 17% from cluster 1 (3 out 18) showed a subthreshold resonance, at a similar resonant frequency of 2.4 ± 0.5 Hz (Figure 2G; Chi-square test = 8.41; *p* = 0.0037). These results strengthen the validity of the clustering classification.

#### Action Potential Waveforms and Spike Train Properties

Cluster 1 neurons present a significantly more depolarized spike threshold (−41.4 ± 0.9 vs. −45.9 ± 1.1 mV, *p* = 0.0039), which may partially counteract the differences in passive membrane properties. Spike waveforms also differed significantly (Figure 3A), with cluster 1 cells showing a smaller spike amplitude (70.6 ± 2.6 vs. 81.4 ± 1.8 mV, *p* = 0.0011), a slower AP rise slope (90.5 ± 3.8 vs. 100.3 ± 3.2 mV.ms^–1^, *p* = 0.0090) and a smaller decay time (1.27 ± 0.09 vs. 1.68 ± 0.14 ms, *p* = 0.0237). The AP was generally followed by a sequence of afterpotentials, which are important feedback mechanisms controlling the AP duration and the refractory period: a fast AHP, followed by a depolarizing afterpotential (detected in 26/50 cells) and then a medium AHP. The fast AHP component was twice as large in cluster 1 relative to cluster 2 (12.3 ± 0.9 vs. 6.6 ± 0.7 mV, *p* < 0.0001), at the expense of the mAHP (2.9 ± 0.4 vs. 6.8 ± 0.8 mV, *p* < 0.0001). This led to an overall AHP of similar amplitude (14.5 ± 0.8 vs. 12.5 ± 0.8 ms, *p* = 0.1323), but with a significantly shorter duration in cluster 1 (25 ± 3 vs. 85 ± 18 ms, *p* = 0.0011). Importantly, we detected no significant difference in the AP duration between the two groups (0.96 ± 0.04 vs. 1.10 ± 0.05 ms, *p* = 0.0533).

In addition, the properties of spike trains evoked by supra-threshold current injections again highlighted strong differences in excitability between the two clusters (Figure 3B): the firing rate at + 40 pA from rheobase was higher in cluster 1 (24 ± 2 vs. 10 ± 1 Hz, *p* < 0.0001). Similarly, the I/O gain, defined as the slope between the average firing frequency and the injected current, was higher in cluster 1 (0.41 ± 0.03 vs. 0.18 ± 0.02 Hz.pA^–1^, *p* < 0.0001). In addition, the early and late spike frequency adaptation indices, characterizing the time-dependent decrease in spike discharge rate under repetitive firing, were about twice smaller in cluster 1 neurons (respectively, 1.3 ± 0.1 vs. 2.3 ± 0.2 and 1.9 ± 0.2 vs. 4.1 ± 0.6, *p* < 0.0001 for both indices). Overall, these results indicate that cluster 2 pyramidal cells are less excitable and display stronger spike frequency adaptation compared to cluster 1 neurons. These conclusions are also supported by the distinct average profiles of the f-I curves (Figure 3B), displaying a higher overall activity of cluster 1 [Linear Mixed Model fixed effect of cluster identity *F*(1, 48.3) = 62.7, *p* < 0.0001] and a stronger gain [fixed effect of interaction between cluster identity and injected current *F*(15, 603.6) = 24.9, *p* < 0.0001]. The stronger adaptation observed in cluster 2 was associated with a higher proportion of cells displaying an initial burst, i.e., a spike doublet (GLMM, fixed effects of cluster identity and injected current *p* < 0.0001). To investigate the interplay between these differences in spike frequency adaptation and excitability, we measured the adaptation ratio (using two different metrics: either using the late SFA index, or the average of the differences between consecutive ISIs in order to minimize the influence of the initial spike doublet) for increasing steps intensity and compared the two clusters at intensities eliciting the same number of spikes. Both measures confirmed a markedly stronger adaptation in cluster 2 [LMM, fixed effect of cluster identity for adaptation ratio *F*(1, 50.6) = 20.19, *p* < 0.0001; fixed effect for cluster identity for mean adaptation ratio *F*(1, 48.9) = 12.336, *p* = 0.001]. In both clusters, the adaptation ratio decreased as the number of spikes in the train increased [LMM, fixed effect of number of spikes in the train: *F*(7, 208) = 35.98, *p* < 0.0001], but the decrease was stronger in cluster 2 [LMM, fixed effect of interaction *F*(7, 208) = 9.23, *p* < 0.0001]. Conversely, in both clusters the mean adaptation ratio did not depend on the number of spikes in the train [LMM, fixed effect of number of spikes *F*(6, 169.8) = 0.243, *p* = 0.9615; fixed effect of interaction: *F*(6, 169.8) = 0.255, *p* = 0.9566]. These results confirm that the difference in spike frequency adaptation between the two clusters is not simply due to their difference in excitability, which would induce a different number of spikes in the train, but probably depends on the distinct activation of specific potassium channels and/or differences in inactivation of sodium channels.

In a subset of neurons (*n* = 36), the spontaneous activity was monitored for 30 s (Figure 3C). The proportion of spontaneously active cells and the mean firing rate were significantly different in the 2 clusters (*p* = 0.0201, chi-square test and *p* = 0.0202, rank-sum test), with cluster 2 cells being mainly silent (2 active neurons out of 20 tested) while about half of cluster 1 cells (7 out of 16 tested) displayed spontaneous activity. Two of these cells had sporadic activity (1 spike in 30 s), while the others displayed a tonic firing (average 11.5 ± 2.3 Hz, *n* = 5, range 6.5–19.2 Hz), with a highly regular pattern (CV range: 0.099–0.21) except for one stuttering cell (CV = 1.37). Among the two active cells of cluster 2, one displayed a low firing irregular pattern (1.4 Hz, CV = 1.02) and the other a sustained regular firing pattern (6.9 Hz, CV = 0.13).

### Different Integration Rules of L2/3 FEF Inputs

**FIGURE 4:**
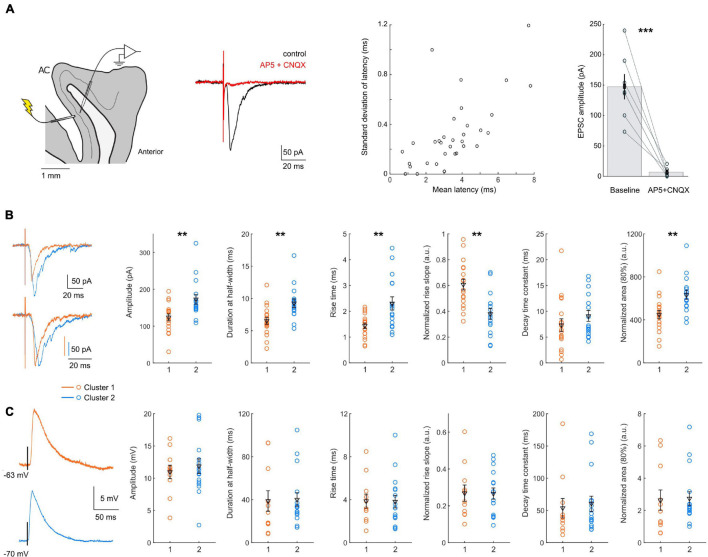
Subthreshold responses to FEF L2/3 stimulation. **(A)** Left: diagram of the experiment configuration, and example EPSCs evoked by layer 2/3 stimulation, under control conditions (black) or in the presence of glutamate blockers (red). Center: scatter plot of the latency and SD of the latency of the responses in each cell tested (*n* = 34). Right: bath application of glutamate transmission blockers suppressed the response to layer 2/3 stimulation. **(B)** Left: example of single EPSCs recorded in cluster 1 (orange) and cluster 2 (blue) neurons, with (bottom) or without (top) amplitude normalization; Right: scatter plots of EPSC properties, with mean and SEM indicated in black. Cluster 1 neurons had EPSCs of smaller amplitude (*p* = 0.0023) and area (*p* = 0.0025), with faster kinetics: a shorter duration (*p* = 0.0018), shorter rise time (*p* = 0.0083) and faster normalized rise slope (*p* = 0.0011). **(C)** Left: example of single EPSPs recorded in cluster 1 (orange) and cluster 2 (blue) neurons. Right: scatter plots of EPSP properties, with mean and SEM indicated in black. No significant difference was detected between the EPSP properties of the two clusters. All data: mean ± SEM. *p < 0.05; **p < 0.01; ***p < 0.001.

We next investigated whether these two groups of cells differentially integrated cortical inputs. For this purpose, we applied electrical stimulation using an electrode placed in layer 2/3 of the FEF slice and analyzed responses in deep-layer pyramidal cells (Figure 4A) in voltage-clamp or current-clamp.

First, the mean and SD latency of all the responses were short (on average: 3.3 ± 0.3 ms, maximum 7.8 ms, and 0.4 ± 0.05 ms, maximum 1.2 ms, *n* = 34), suggesting a monosynaptic connection. We next verified that the responses were glutamatergic, by bath application of AMPA and NMDA glutamate receptor blockers (CNQX 10 μM and AP-5 50 μM). We observed a reduction of the EPSC amplitude by 94.6 ± 2.5% (*p* < 0.001, *n* = 7), effectively suppressing the response and confirming its glutamatergic nature (Figure 4A).

We then compared EPSC characteristics between the two clusters (Figure 4B and Table 4, *n* = 18 for cluster 1; *n* = 16 for cluster 2). The latency and the latency SD of the response were similar in both clusters (average latency: 3.2 ± 0.5 ms vs. 3.24 ± 0.3 ms, *p* = 0.6169; latency SD: 0.44 ± 0.07 ms vs. 0.37 ± 0.05 ms, *p* = 0.9313). Smaller EPSCs were recorded in cluster 1 neurons (amplitude: 124 ± 9 pA, *n* = 18 vs. 173 ± 13 pA, *n* = 16, *p* = 0.0023; area: 818 ± 7 vs. 1,109 ± 72, *p* = 0.0136), with a shorter duration at half-width (6.5 ± 0.5 ms vs. 9.3 ± 0.7 ms, *p* = 0.0018). The rise time of non-normalized EPSCs was shorter for cluster 1 neurons (1.4 ± 0.1 ms vs. 2.3 ± 0.3 ms, *p* = 0.0083), confirmed by a higher rising slope on responses normalized by their amplitude (0.61 ± 0.04 ms^–1^ vs. 0.38 ± 0.04 ms^–1^, *p* = 0.0011). No difference was detected in EPSCs decay time constant, obtained by fitting the decay phase of the normalized EPSCs with a single exponential (7.3 ± 1.2 ms vs. 9.1 ± 1.1 ms, *p* = 0.1841). The area of normalized EPSCs also differed, with a smaller area for cluster 1 cells (449 ± 38 vs. 634 ± 43, *p* = 0.0025).

**TABLE 4:** EPSCs and EPSPs properties in response to L2/3 FEF stimulation.

EPSC	Cluster 1 (*n* = 18)	Cluster 2 (*n* = 16)	*p*-value (Mann-Whitney)
Latency (ms)	3.2 ± 0.5	3.4 ± 0.3	*p* = 0.6169
Latency SD (ms)	0.44 ± 0.07	0.37 ± 0.05	*p* = 0.9313
Amplitude (pA)	123.8 ± 9.2	172.8 ± 13.3	**p = 0.0023
Area 80% (10^4^ pA.ms)	5.4 ± 0.7	10.2 ± 0.8	***p < 0.001
Normalized area 80%	449 ± 38	634 ± 43	**p = 0.0025
Duration at half-width (ms)	6.5 ± 0.5	9.3 ± 0.7	**p = 0.0018
Rise time 20–80% (ms)	1.4 ± 0.11	2.30 ± 0.26	**p = 0.0083
Normalized rise slope	0.61 ± 0.04	0.38 ± 0.04	**p = 0.0011
Normalized decay time constant (ms)	7.3 ± 1.2	9.1 ± 1.1	*p* = 0.1841

**EPSP**	**Cluster 1 (*n* = 11)**	**Cluster 2 (*n* = 16)**	

Latency (ms)	2.4 ± 0.4	3.0 ± 0.3	*p* = 0.1323
Latency SD (ms)	0.58 ± 0.07	0.46 ± 0.07	*p* = 0.0887
Amplitude (mV)	11.0 ± 1.0	11.9 ± 1.1	*p* = 0.9803
Area 80% (10^4^ mV.ms)	2.8 ± 0.7	3.1 ± 0.5	*p* = 0.5373
Normalized area 80%	2,600 ± 600	2,700 ± 400	*p* = 0.5704
Duration at half-width (ms)	38.8 ± 9.5	40.1 ± 6.4	*p* = 0.6044
Rise time 20–80% (ms)	3.9 ± 0.7	3.8 ± 0.6	*p* = 0.9803
Normalized rise slope	0.27 ± 0.04	0.27 ± 0.03	*p* = 0.9410
Normalized decay time constant (ms)	53 ± 15	60 ± 12	*p* = 0.5053

All data: mean ± SEM. *p < 0.05; **p < 0.01; ***p < 0.001.

We also compared EPSP waveforms recorded in current-clamp mode (Figure 4C and Table 4, *n* = 11 for cluster 1; *n* = 16 for cluster 2). Similar to EPSCs, EPSP latencies and latency SD did not differ between cluster 1 and 2 (mean latency: 2.4 ± 0.4 ms vs. 3.0 ± 0.3 ms, *p* = 0.1323; latency SD: 0.58 ± 0.07 ms vs. 0.46 ± 0.07, *p* = 0.0887). Interestingly, the differences between cluster 1 and cluster 2 EPSCs were not detected in EPSPs. Indeed, neither the amplitude (11.0 ± 1.0 mV vs. 11.9 ± 1.1 mV, *p* = 0.9803) nor the kinetics of the EPSPs (duration at half-width: 38.8 ± 9.5 ms vs. 40.1 ± 6.4 ms, *p* = 0.6044; rise time: 3.8 ± 0.7 ms vs. 3.8 ± 0.6 ms, *p* = 0.9803) was significantly different.

**FIGURE 5:**
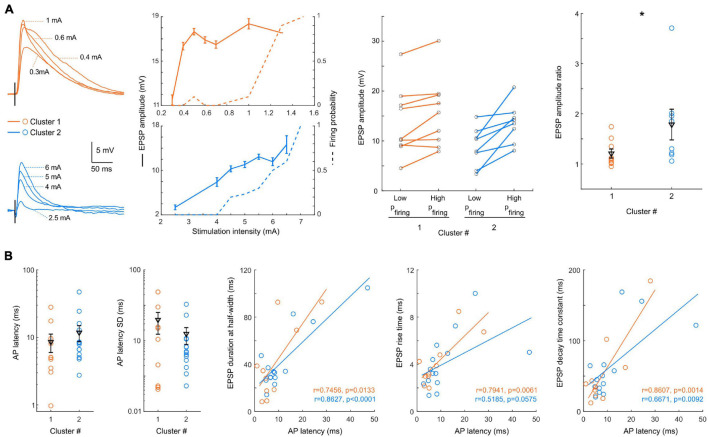
Input/output and suprathreshold response to L2/3 stimulation. **(A)** Left: example of EPSPs (smoothed average of 10 stimulations excluding spiking events) recorded in a cluster 1 (orange) and a cluster 2 (blue) neuron in response to increasing stimulation intensity of layer 2/3, with the EPSP amplitude and probability of firing plotted as a function of stimulation amplitude for these example neurons. Center: for each neuron, the amplitude of EPSPs is plotted for a low stimulation intensity (last intensity before spiking is evoked) and a high stimulation intensity (spiking probability of 0.7–0.9). Right: the ratio between EPSP amplitudes at high/low stimulation amplitudes is significantly lower in cluster 1 neurons (*p* = 0.04). **(B)** From left to right: mean latency, and SD of the latency of spikes evoked by suprathreshold stimulation. Significant correlations are observed between the spike latency in suprathreshold responses and the kinetics (duration at half-width, rise time and decay time constant) of subthreshold EPSPs. All data: mean ± SEM. *p < 0.05.

We next analyzed the responses to increasing cortical stimulation strength (Figure 5A). We observed two main types of integration patterns: about half of the cells progressively increased their probability of firing a spike (*n* = 9 out of 19), while the other half switched abruptly from sub- to suprathreshold responses (*n* = 10 out of 19). Due to the difficulty of normalizing EPSPs responses by the stimulation current because recordings were done with different electrophysiological set-ups and stimulation electrodes, and on different slices, we chose to select the amplitude of the responses to the last stimulation current never evoking an action potential and the amplitude of the responses evoking an action potential with a 0.7–0.9 probability. Interestingly, the ratio between the amplitudes of these two responses was significantly different between the two clusters (*p* = 0.0464, Figure 5A), with a larger gain for cluster 2 pyramidal cells (1.2 ± 0.09, *n* = 9 vs. 1.8 ± 0.3, *n* = 8), while the EPSPs amplitudes were not significantly different (13.7 ± 2.3 mV, *n* = 9 vs. 8.8 ± 1.3 mV, *n* = 9, *p* = 0.1672; and 15.7 ± 2.3 mV, *n* = 8 vs. 13.6 ± 1.4 mV, *n* = 8, *p* = 0.6730). For suprathreshold events, the mean latency and jitter (latency SD) of evoked spikes were similar in the two clusters (cluster 1: *n* = 10; cluster 2: *n* = 14) (Figure 5B) (*p* = 0.2658 and *p* = 0.7696, respectively). Interestingly, in both clusters a correlation was observed between the spike latency in suprathreshold responses and the kinetics of subthreshold EPSPs (Figure 5B). In particular, the strong correlation with the EPSP duration at half-width segregated the neurons into two distinct groups: one with short EPSPs and fast-spiking response, and the other with slower EPSPs and evoked spikes, both groups being represented in the two clusters.

**FIGURE 6:**
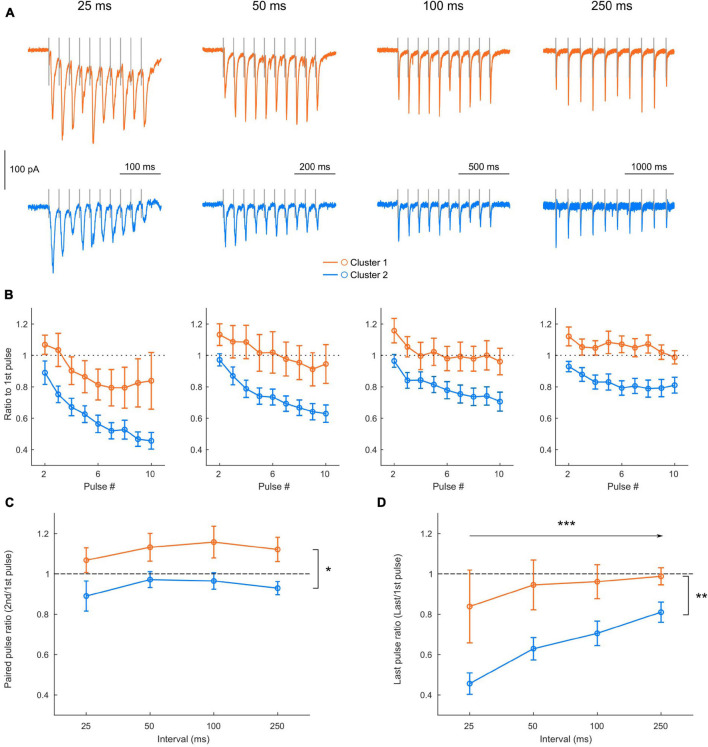
Clusters display different short-term plasticity properties. **(A)** Example of EPSCs recorded in a cluster 1 (orange) and a cluster 2 (blue) neuron in response to trains of 10 stimulations, for increasing stimulation intervals. **(B)** Average amplitude of the EPSCs along the train (starting from the second EPSC) in cluster 1 (orange) and cluster 2 (blue) neurons, normalized in each neuron by the amplitude of the first EPSC of the train, for each stimulation interval (see 3-way ANOVA results in Results). **(C,D)** Influence of the stimulation interval on the first paired pulse ratio [**C:** EPSC2/EPSC1; 2-way repeated measures ANOVA, effect of cluster *F*(1, 20) = 7.23, *p* = 0.0141; Effect of interval *F*(3, 60) = 1.7040, *p* = 0.1758; Interaction *F*(3, 60) = 0.0713, *p* = 0.9751] and on the last pulse ratio [**D:** EPSC10/EPSC1; 2-way repeated measures ANOVA, effect of cluster *F*(1, 20) = 8.20, *p* = 0.0096; Effect of interval *F*(3, 60) = 10.01, *p* < 0.0001; Interaction *F*(3, 60) = 1.66, *p* = 0.1849]. All data: mean ± SEM. *p < 0.05; **p < 0.01; ***p < 0.001.

Finally, we investigated short-term plasticity properties of the responses to L2/3 FEF inputs in the 2 clusters (cluster 1: *n* = 8 neurons, cluster 2: *n* = 14 neurons) by applying trains of 10 pulses at various interpulse intervals (25, 50, 100, and 250 ms) (Figure 6A). Normalizing the EPSC amplitude at each successive pulse to the EPSC amplitude at the first one (Figure 6B) revealed that both clusters adapted their response to the train, with short-term facilitation or depression indicated by a ratio superior or inferior to 1, respectively. A 3-way ANOVA [Cluster identity X Stimulation interval X pulse number, repeated measures in each neuron; main effect of pulse number: *F*(8, 160) = 40.168, *p* < 0.0001], revealed an effect depending on the interval [main effect of interval: *F*(3, 60) = 12.070, *p* < 0.0001] and an effect depending on the clusters [main effect of cluster identity: *F*(1, 20) = 8.98, *p* = 0.0071]. Indeed, after little change in the initial paired-pulse ratio (Figure 6C), cluster 2 neurons displayed a strong depression instated along the train, more pronounced for shorter stimulation intervals (Figure 6D). Conversely, cluster 1 neurons displayed a facilitation in the initial paired-pulse ratio, independently of the stimulation interval (Figure 6C), which degraded along the train and turned into a depression for short stimulation intervals (Figure 6D). These results suggest that the two clusters identified using cell-intrinsic properties differentially integrate afferent signals from superficial cortical layers.

### Validity of the Classification and Analysis for Four Clusters

The segregation obtained for two clusters was robust since only one mismatch could be detected when modifying the number of principal components used in the k-means algorithm or when performing the clustering directly on the whole normalized dataset (Table 2). In addition, when performing k-means classification on scrambled data, on average 7 electrophysiological parameters reached significance level (with only 2 below 0.001 and 2 between 0.01 and 0.05), while in our dataset we obtained high significance levels for 12 parameters out of 18 (with 8 below 0.001 and 4 between 0.01 and 0.05).

Since four clusters also appeared to be an optimal classification for some indicators, we also calculated the mean electrophysiological properties related to each cluster (Table 5), as well as the indices quantifying the robustness of the clustering method for four clusters (Tables 1–5). Importantly, we retrieve within the four clusters two subgroups with high resistance, more depolarized RMP and high excitability (cluster 1 and 3, *n* = 10 and 8 neurons, respectively) and two subgroups with low resistance, more hyperpolarized RMP and low excitability (cluster 2 and 4, *n* = 22 and 10 neurons, respectively). Furthermore, one can note that cluster 1 has a faster decay of its action potential (37.2 ± 3.3), twice faster than cluster 3 (81.1 ± 8.5), while cluster 3 is characterized by the large amplitude of its fast AHP component (16.8 ± 1.2), about twice as large compared to the other clusters. Cluster 2 distinguishes itself by a very hyperpolarized AP threshold (−46.6 ± 1.1 mV). Cluster 4 adapts its firing pattern far more strongly compared with all three clusters, as visible in both early and late SFA indices (3.3 ± 0.4 and 6.4 ± 1, respectively), has a particularly low I/O gain and firing rate at + 40 pA from rheobase, and hence presents the lowest excitability of all subgroups. In addition, subthreshold resonance is strictly observed in the two less excitable subgroups (cluster 2: with 11 out of 17 cells and cluster 4: 3 out of 8 cells, Chi-square = 11.01; *p* = 0.0117).

**TABLE 5:** Electrophysiological properties of four clusters.

Mean ± SEM	Cluster 1 (*n* = 10)	Cluster 2 (*n* = 22)	Cluster 3 (*n* = 8)	Cluster 4 (*n* = 10)	*p*-value (Kruskal-Wallis)
Resting membrane potential (mV)	−62.6 ± 1.4	−65.8 ± 0.7	−60.9 ± 3.0	−68.7 ± 1.2	χ^2^ = 10.36; *p* = 0.0157 *1–4
Membrane resistance (MΩ)	488 ± 86	224 ± 27	467 ± 100	125 ± 14	χ^2^ = 22.9; *p* < 0.0001 ***1–4; **3–4; *1–2
Membrane time constant (ms)	39 ± 7	24 ± 3	28 ± 4	26 ± 2	χ^2^ = 3.4; *p* = 0.34
Rheobase (pA)	10 ± 4	42 ± 9	14 ± 5	89 ± 19	χ^2^ = 18.1; *p* = 0.0004 ***1–4; **3–4
Sag Index (%)	16 ± 4	21 ± 2	22 ± 4	25 ± 3	χ^2^ = 4.7; *p* = 0.20
Rebound Index (%)	−0.9 ± 8	34 ± 13	9 ± 9	88 ± 20	χ^2^ = 18.5; *p* = 0.0003 ***1–4; **3–4
AP threshold (mV)	−40.6 ± 1.2	−46.6 ± 1.1	−38.2 ± 1.4	−43.9 ± 1.0	χ^2^ = 19.5; *p* = 0.0002 ***2–3; *1–2; 3–4
AP amplitude (mV)	61.5 ± 4.0	83.7 ± 1.6	66.6 ± 3.2	80.0 ± 1.5	χ^2^ = 29.5 *p* < 0.0001 ***1–2; 2–3; *1–4; 3–4
AP duration at half-width (ms)	1.17 ± 0.09	1.02 ± 0.04	0.82 ± 0.06	1.08 ± 0.08	χ^2^ = 9.3; *p* = 0.0253 *1–3
AP rise slope (mV.ms^–1^)	71.7 ± 4.7	106.0 ± 2.3	86.7 ± 3.9	101.7 ± 4.6	χ^2^ = 29.3; *p* < 0.0001 ***1–2; **1–4; 2–3
AP decay slope (mV.ms^–1^)	37.2 ± 3.3	60.5 ± 3.2	81.1 ± 8.5	57.4 ± 6.3	χ^2^ = 19.4; *p* = 0.0002 ***1–3; **1–2
AHP amplitude (mV)	10.7 ± 1.3	13.6 ± 0.7	17.4 ± 1.4	13.2 ± 1.3	χ^2^ = 11.5; *p* = 0.0094 **1–3
fAHP amplitude (mV)	7.5 ± 1.0	9.0 ± 0.9	16.8 ± 1.2	7.0 ± 1.3	χ^2^ = 17.8; *p* = 0.0005 **1–3; 2–3;3–4
ADP amplitude (mV)	0.05 ± 0.05	0.52 ± 0.1	2.2 ± 0.5	1.7 ± 0.6	χ^2^ = 14.7; *p* = 0.0021 **1–3; *1–4
mAHP amplitude (mV)	3.3 ± 0.7	5.1 ± 0.8	2.2 ± 0.6	7.9 ± 1.1	χ^2^ = 12.4; *p* = 0.006 **3–4; *1–4
AHP duration (ms)	25.2 ± 4.1	35.4 ± 4.9	21.9 ± 4.2	148 ± 35	χ^2^ = 11.3; *p* = 0.0104 *1–4; 3–4
Firing rate at + 40 pA from rheobase (Hz)	24 ± 2	15 ± 1	27 ± 3.4	8 ± 1	χ^2^ = 24.8; *p* < 0.0001 ***1–4; 3–4; *1–2; 2–3
Early spike frequency adaptation	1.6 ± 0.1	1.5 ± 0.1	1.2 ± 0.1	3.3 ± 0.4	χ^2^ = 26.3; *p* < 0.0001 ***2–4; 3–4; *1–4
Late spike frequency adaptation	2.6 ± 0.4	2.1 ± 0.2	1.5 ± 0.1	6.4 ± 1	χ^2^ = 27.6; *p* < 0.0001 ***2–4; 3–4; *1–3
I/O gain (Hz.pA^–1^)	0.41 ± 0.04	0.27 ± 0.03	0.48 ± 0.07	0.15 ± 0.03	χ^2^ = 21.4; *p* < 0.0001 ***3–4; **1–4; *2–3
Spontaneous frequency (Hz)	6.8 ± 3.0	1.7 ± 1.0	5.8 ± 3.7	0	χ^2^ = 7.3; *p* = 0.062

All data: mean ± SEM. *p < 0.05; **p < 0.01; ***p < 0.001.

## Discussion

Our recordings of intracellular electrophysiological properties of deep-layer pyramidal cells in FEF of macaque monkeys allowed us to distinguish two major types of regular-spiking neurons. On the one hand, the first group consists of cells with an increased excitability (depolarized RMP, higher Ri, lower rheobase, higher spontaneous and current-evoked activity, stronger I/O gain, and weaker spike frequency adaptation), with fewer resonant cells. These cells responded to superficial layer stimulation with smaller but faster EPSCs, and an initial facilitation for paired stimulations. On the other hand, the second cell type is characterized by a decreased excitability (hyperpolarized RMP and lower Ri, higher rheobase, lower spontaneous and current-evoked activity, weaker I/O gain and stronger adaptation), associated with a higher proportion of cells displaying a preferred resonant frequency at ∼2 Hz, and a higher proportion of cells initiating their spike trains with a doublet. They responded to superficial layer stimulation with stronger but slower EPSCs, and a progressive depression in response to repeated stimulations, in particular for short-time intervals.

In our sample of cells, FEF contained nearly half of each population (*n* = 26 for cluster 1 and *n* = 24 for cluster 2 out of 50 cells). Interestingly, these two types of regular-spiking pyramidal cells with notably different degree of spike adaptation have been reported in the L5 of monkey or rat prefrontal cortex or in cat association cortices (Nuñez et al., 1993; Dégenètais et al., 2002; Chang and Luebke, 2007). In addition, in these studies, a small proportion of intrinsic burst firing cells, characterized by an initial all-or-none burst at depolarizing steps, and also fast-adapting pyramidal neurons, which present a depolarizing plateau following an initial train of spikes were observed. However, we did not record any intrinsic burst firing cells or regular-spiking fast-adapting cells in FEF deep layers, which may be due to our limited sample or specificities of the FEF. In addition to pyramidal neurons, we recorded and briefly characterized three fast-spiking interneurons (Supplementary Figure 2 and Supplementary Table 2).

How can we relate these two main types of intracellular properties to the functional diversity of FEF neurons? Currently, correlations between the functional properties of FEF neurons and their anatomical and electrophysiological signatures have not been elucidated. Pioneer studies have reported three main types—visual, movement and visuo-movement neurons (Bruce and Goldberg, 1985; Schall, 1991). Yet these classes are not strictly distinct and rather form a continuum, with diverse patterns of spike rate modulation visible during a typical memory-guided saccade task (Lowe and Schall, 2018). In this perspective, the specificities of each cortical layer need to be considered, especially now that recent evidence starts to unveil the computations performed by supra-granular and deep layers, respectively (Heinzle et al., 2007; Bastos et al., 2018; Yu et al., 2019). In particular, FEF L5 pyramidal cells have been distinguished by their projecting targets, with cortico-pontine neurons carrying in half-cases movement-related information (Segraves and Goldberg, 1987; Segraves, 1992), and corticotectal neurons projecting to the superior colliculus carrying cognitive and sensory-related information (Sommer and Wurtz, 2000). It would be far too simplistic to try mapping our two clusters to these highly heterogeneous functional categories. However, we can still draw hypotheses between the characteristics found by our classification and the distinct activity (transient or sustained) and response patterns (with or without a delay) reported within these three main categories.

On the one hand, cluster 1 neurons with their higher excitability profile appear as a preferred candidate over less excitable regular-spiking pyramidal neurons to encode precise and persistent information, similarly to what was concluded with the help of a computational model in the retrosplenial cortex (Brennan et al., 2020). Spike frequency adaptation has also been shown to destabilize persistent firing (Carter and Wang, 2007). Thus, the features of cluster 1 neurons in the FEF may facilitate the production of tonic discharge in the continuous presence of inputs such as during fixation (Izawa and Suzuki, 2014), or ramping sustained activity when an accumulation of evidence on the sensory target position is being processed, as well as in persistent firing linked to attention (Moore and Fallah, 2001; Armstrong et al., 2009) or in the slow return to baseline following the saccade (Hanes et al., 1995; Lowe and Schall, 2018). On the other hand, cluster 2 pyramidal cells would require stronger inputs to produce an output spiking response, because of their higher rheobase and lower input resistance. Yet, their output may then be more reliably transmitted to downstream targets due to the higher propensity of these cells to spike high-frequency doublets. In addition, due to their high spike-frequency adaptation, their responses may remain clipped to the time of the stimulus, which could be either the visual target or the saccade command. They could thus become active at the end of the hypothesized “winner-take-all competition process” that may be at stake during the saccade generation process (Itti and Koch, 2000; Thompson and Bichot, 2005). Thus, cluster 2 neurons could contribute preferentially to movement generation or participate in feedforward target sensory processing, while cluster 1 neurons would rather provide feedback information to supra-granular layers, controlling for example working memory maintenance (Bastos et al., 2018).

In addition to differences in intrinsic properties, significant differences in evoked EPSC kinetics were observed between the two clusters. Yet these differences were no longer visible in current-clamp recordings at the somatic level. This may be explained “passively” by the high resistance of cluster 1 cells, which may counteract the smaller elicited currents evoked under L2/3 stimulation or it could be linked to homeostatic processes, that actively lowered the excitatory drive of those intrinsically more excitable neurons (Debanne et al., 2019). In addition, these results cannot exclude differential integration patterns at the dendritic level, especially given that dendrites and the soma appear more strongly compartmentalized in primates (Beaulieu-Laroche et al., 2018).

Interestingly, we also found a wide range of mean latency and latency SD when a single action potential was evoked by L2/3 stimulation, with overlaps between the two clusters, that could be correlated to EPSP kinetics. Our results showed that the highest temporal precision of the spiking response relative to the stimulation time was found in cells whose EPSPs have a short halfwidth, rise time or decay time constant, in agreement with model predictions (Rodriguez-Molina et al., 2007). Importantly, the variability in the spike latency could endow neurons with different functional roles. Indeed, if the nature of L2/3 inputs has not been elucidated, the computational model of Heinzle et al. (2007) provide useful hypotheses, suggesting that L2/3 neurons transform the visual saliency map, carried by layer 4 neurons, into an attentional signal, sending the position of the selected target to L5 neurons, while also possibly generating a motor plan due to feedback connections. In addition, *in vivo* recordings have shown that the peak activity of visual or movement-related neurons varied across categories (Lowe and Schall, 2018). One could hypothesize that neurons presenting short-latency evoked spikes would be preferentially involved in the generation of the pre-saccadic bump of activity, while neurons with longer-latency spikes may be associated with less “clipped” activity relative to the visual input or the saccade production.

In addition, the short-term plasticity observed between L2/3 and deep-layer excitatory connections is dominated by depression for short-time intervals, as observed in other cortical areas across different species. This general property of the cortical microcircuit has been described as a means for gain control, producing equal post-synaptic responses to rapidly or slowly firing afferents, and generating an enhanced sensitivity to fast changes in presynaptic firing rate (Abbott et al., 1997). This phenomenon may be particularly crucial on the one hand for some movement-related FEF neurons that generate a punctual and transient response, either before or immediately after the saccade. Such activity may thus be aided by the stronger depression observed in cluster 2 neurons which also strongly adapt their firing rates. On the other hand, cluster 1 neurons, which present smaller short-term depression, with even an initial facilitation at the start of repeated L2/3 stimulation trains, may transmit action potentials more reliably during bursts of activity. This mechanism could act in synergy with their small firing rate adaptation, to maintain tonic discharge patterns, such as in the case of fixation neurons (Izawa and Suzuki, 2014) or during delays in which attention or memory-related information needs to be maintained (Bruce and Goldberg, 1985; Sommer and Wurtz, 2000). Yet, several questions remain unresolved relative to the synaptic properties of L2/3-L5 fast-spiking interneurons and L5 recurrent excitatory connections, that could also contribute to shaping persistent activity patterns at the network level (Yoon et al., 2020).

In our recordings, 40% of pyramidal neurons displayed subthreshold resonance at about 2 Hz, with a significant majority present in the low-resistance cluster. This proportion is similar to the one found in a recent study on human cortical pyramidal cells (Moradi Chameh et al., 2021). If such subthreshold resonance is usually associated with the expression of Ih currents (Beaulieu-Laroche et al., 2018; Moradi Chameh et al., 2021), we did not find significant correlations between the existence of a large sag voltage and low-frequency resonance when considering all resonant and non-resonant neurons. This may indicate that additional ionic currents such as the persistent Na^+^ current may also drive the subthreshold resonance observed here. In the presence of high levels of fluctuations, as observed *in vivo* in cortical circuits, such subthreshold resonance may also turn into or at least favor a firing-rate resonance (Brunel et al., 2003), making cluster 2 neurons likely candidates in participating to theta-coupling with V4 during visual search (Yan and Zhou, 2019) as well as to higher gamma coupling during attentional tasks (Gregoriou et al., 2009). As previously reported, few neurons in layer 5 of FEF project to visual cortex (V4 or inferotemporal area) and very few if any of these neurons have axons that also terminate in the superior colliculus (Pouget et al., 2009). These results have been seen as strong suggestions that deep neurons of FEF that project to visual cortex are to be considered a feedforward or intermediate type of pathways. In the same vein, we consider that these data are consistent with the hypothesis that the signal in extrastriate cortex received from FEF relates to target selection and not saccade planning and could be sent by a majority of cluster 2 neurons, also in agreement with their electrophysiological properties as discussed above. Altogether, these frequency couplings may optimize the postsynaptic impact between FEF and V4 (Gregoriou et al., 2009) or between FEF and anterior cingulate cortices during sensorimotor mapping (Babapoor-Farrokhran et al., 2017).

Notably, our clustering analysis did not highlight a difference in spike width, as reported by some studies using extracellular recordings. Yet, this feature used for classifying movement and visual neurons vs. visuo-movement neurons, which would have the thinnest spikes (Cohen et al., 2009; Ding and Gold, 2012; Thiele et al., 2016), still remain debated, as a more recent study report no significant difference (Lowe and Schall, 2018). In addition, correlations between intracellular recordings and extracellular waveforms should be subjected to a careful interpretation: only about 50% of the variance could be explained by the intracellular features according to a recent study (Xu and Baker, 2018), while external factors such as the distance to the recorded cells and the filtering properties of the extracellular matrix need also to be considered (Nelson et al., 2013).

A future step in the characterization of FEF neuronal subpopulation would be to examine their morphological features and whether they segregate with electrophysiological clusters. First of all, this would allow to rigorously confirm the pyramidal nature of all recorded neurons. Indeed, our spike shape criteria to exclude interneurons cannot discriminate broad spiking interneurons, such as VIP cells—though from proportion alone, combined with visual targeting of pyramidal soma shapes in slices, it is highly unlikely that their number would populate a cluster representing 50% of our sample. Secondly, a morphological quantification would be particularly relevant since two groups of L5 pyramidal cells, retrogradely labeled by horseradish peroxidase injections into the superior colliculus, have previously been distinguished based on the size of their soma (Fries, 1984), a morphological feature that can be related to the input resistance, for which our two clusters strongly differ. In addition, differences in axonal conduction times have been reported (Segraves and Goldberg, 1987; Segraves, 1992) with fixation and movement neurons having longer and shorter conduction times, respectively. These results could potentially map our electrophysiological distinctions, since on the one hand, cluster 1 cells, which we hypothesize to be in part fixation neurons due to their discharge pattern, have a high input resistance, and could thus have a smaller soma and a narrower axon, with longer conduction times. On the other hand, cluster 2 neurons, that could send target or saccade signaling, would be larger and have shorter conduction times, because of their high input resistance.

If our electrophysiological recordings targeted FEF L5, one cannot exclude that a minority of recorded neurons were situated at the border between L5/L6. More importantly, the origin of our two clusters could emerge from the existence of gradients within L5, determined for instance by soma depth, projection target and/or dendritic complexity. Interestingly, the considerable electrophysiological differences observed within our sample echoes the large variability reported in human neocortical L5 pyramidal cells (Moradi Chameh et al., 2021), that could partially be explained by a gradient in dendritic complexity (with thick or thin-tufted pyramidal cells at the two extremes).

Some precautions should be taken into consideration for comparing our *in vitro* electrophysiological results with *in vivo* recordings. It thus remains to strengthen the existence of such neuronal clusters using *in vivo* intracellular recordings in non-anesthetized primates. Indeed, we examined the neuronal properties *in vitro* in brain slices maintained at 34°C, thus 3°C below physiological temperature, to increase the viability of the brain slices, but this is known to affect AP kinetics or spiking frequency (Thompson et al., 1985). Nevertheless, if we can expect modified absolute values for intrinsic and active membrane properties for *in vivo* conditions, the belonging of FEF deep layer pyramidal cells to at least two clusters should be confirmed since most of membrane properties evolve linearly with decreasing temperatures, at least in the 37–33°C range (Thompson et al., 1985). Also, the dendritic tree damage caused by slice preparation and the choice of ionic concentrations can influence the membrane properties. In particular, lower calcium concentration can modify the propensity for generating high-frequency spike bursts (Brumberg et al., 2000), which were not visible in our recordings. Finally, we used square current steps to standardize experimental conditions and to extract membrane properties and repetitive firing features that are critical and lacking for modeling studies. Nevertheless, such responses are not readily translatable into *in vivo* firing characteristics; the links we draw between the data from each preparation can only be tentative, and would need to be tested using stimulation protocols that better mimic task-related activity (or conclusively investigated using *in vivo* patch-clamp).

To conclude, the ability to distinguish types of neurons in FEF is necessary to understand whether the visual to motor transformation occurs within or across distinct neuron types. We found that intracellular properties of deep-layer pyramidal cells in FEF of macaque monkeys allow classifying two majors cell types. These results are important to better account for the existence of a functional micro-circuit playing a key role in sensorimotor transformation within the FEF.

## Data Availability Statement

The original contributions presented in the study are included in the article/Supplementary Material, further inquiries can be directed to the corresponding author/s.

## Ethics Statement

The animal study was reviewed and approved by the European Community Council Directives of 1986 (86/609/EEC) and the NIH Guide for the Care and Use of Laboratory Animals and were approved by the French Animal Ethics Committee of INSERM.

## Author Contributions

PP, LV, MV, and CP: conceptualization, writing, review, and editing. CP, MV, CB-B, VG, VP, YC, AM, SP, SV, HX, and LV: investigation and analysis. PP and LV: supervision. All authors contributed to the article and approved the submitted version.

## Conflict of Interest

The authors declare that the research was conducted in the absence of any commercial or financial relationships that could be construed as a potential conflict of interest.

## Publisher’s Note

All claims expressed in this article are solely those of the authors and do not necessarily represent those of their affiliated organizations, or those of the publisher, the editors and the reviewers. Any product that may be evaluated in this article, or claim that may be made by its manufacturer, is not guaranteed or endorsed by the publisher.

## Supplementary Material

Supplementary Figure 1 R_series_ does not segregate with cluster identity. **(A)** R_series_ scatterplots of neurons in each cluster, with mean and SEM indicated in black. For both two (left) or four (right) clusters, the difference between groups is non-significant (left: Mann-Whitney test, *p* = 0.0555; right: Kruskal-Wallis test, *p* = 0.101). **(B)** R_series_ scatterplots with each parameter of the PCA that is significantly different between the two clusters. The correlation coefficient and *p*-value (Pearson’s correlation), calculated with all neurons (clusters pooled) is indicated above each graph.

Supplementary Figure 2 Fast spiking interneurons recorded in FEF Layer 5. Electrophysiological response of a fast spiking interneuron to current step injections (left: from −100 pA, 10 pA-steps up to rheobase; center: +40 pA above rheobase; right: I–V curve).
