# Supplementary material for: Intracellular Properties of Deep-Layer Pyramidal Neurons in Frontal Eye Field of Macaque Monkeys
Source: Front Synaptic Neurosci. 2021 Sep 21;13:725880. doi: 10.3389/fnsyn.2021.725880 (PMC8490863; doi:10.3389/fnsyn.2021.725880)
Supplement: Supplementary Figure 1 — Rseries does not segregate with cluster identity. (A) Rseries scatterplots of neurons in each cluster, with mean and SEM indicated in black. For both two (left) or four (right) clusters, the difference between groups is non-significant (left: Mann-Whitney test, p = 0.0555; right: Kruskal-Wallis test, p = 0.101). (B) Rseries scatterplots with each parameter of the PCA that is significantly different between the two clusters. The correlation coefficient and p-value (Pearson’s correlation), calculated with all neurons (clusters pooled) is indicated above each graph. [file Data_Sheet_1.docx]

Intracellular properties of deep-layer pyramidal neurons in Frontal Eye Field of macaque monkeys

Piette C^1^, Vandecasteele M^1^, Bosch-Bouju C^1^, Goubard V^1^, Paillé V^1^, Cui Y^1^, Mendes A^1^, Perez S^1^, Valtcheva S^1^, Xu H^1^, Pouget P^2*^ and Venance L^1*^

**Supplementary Material**


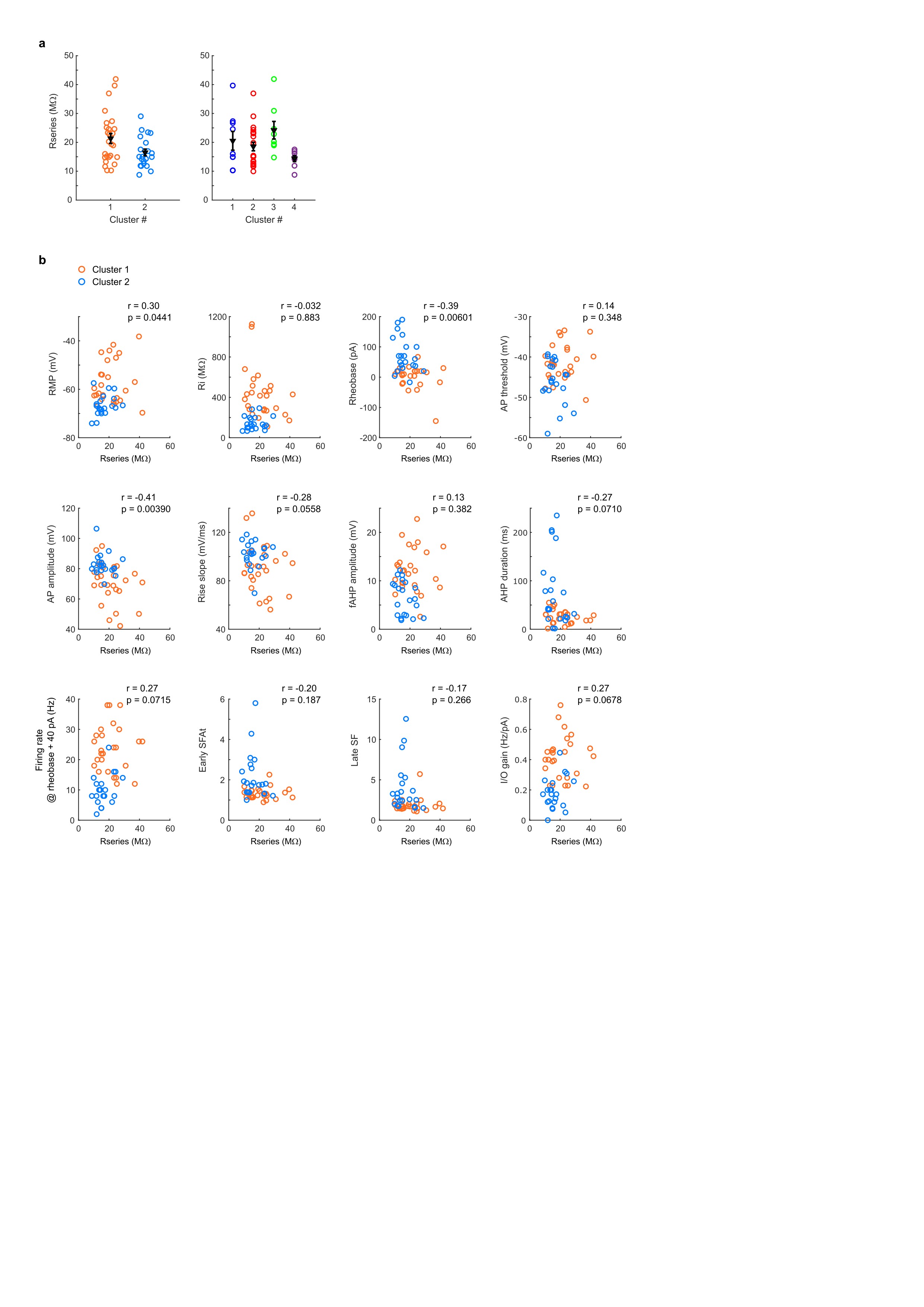


**Supplementary Figure 1: R_series_ do not segregate with cluster identity.**

**a.** R_series_ scatterplots of neurons in each cluster, with mean and SEM indicated in black. For both two (left) or four (right) clusters, the difference between groups is non-significant (left: Mann-Whitney test, p=0.0555; right: Kruskal-Wallis test, p=0.101). **b.** R_series_ scatterplots with each parameter of the PCA that is significantly different between the two clusters. The correlation coefficient and p-value (Pearson’s correlation), calculated with all neurons (clusters pooled) is indicated above each graph.

**
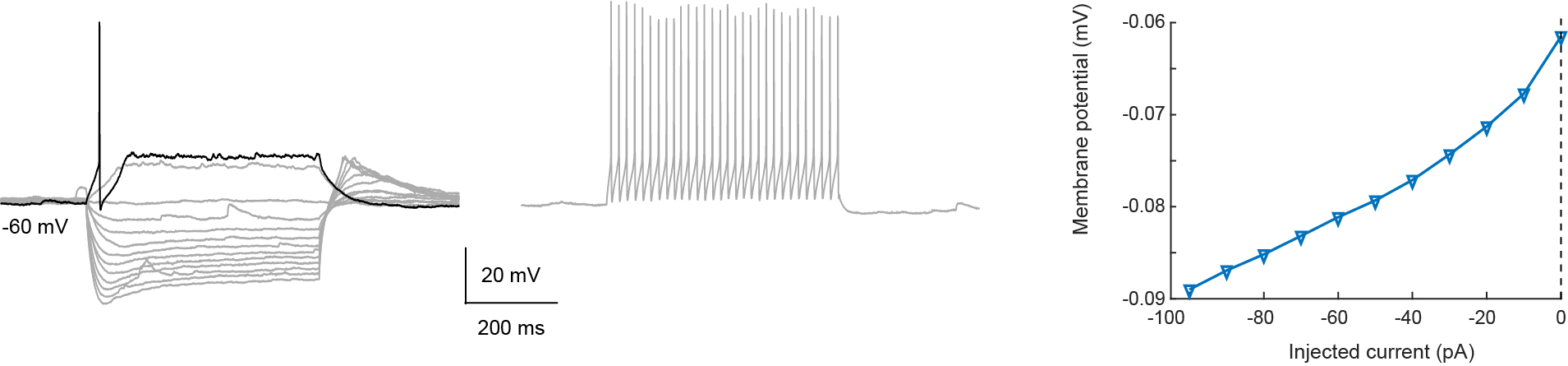
**

**Supplementary Figure 2: Fast spiking interneurons recorded in FEF Layer 5.**

Electrophysiological response of a fast spiking interneuron to current step injections (left: from -100 pA, 10 pA-steps up to rheobase; center: +40 pA above rheobase; right: I-V curve).

**Supplementary Table 1:**

| # Id | Cluster | PCA/General Properties (B=biocytin) | Zap analysis | Spontaneous activity | EPSC  (P=Pharma  cology) | EPSP | I/O gain | EPSP correlations | Paired pulse | R_series_ |
| --- | --- | --- | --- | --- | --- | --- | --- | --- | --- | --- |
|  |  | Fig1b-e,  2a-b, 3a-b | Fig 2c-g | Fig 3c | Fig 4a-b | Fig 4c | Fig 5a | Fig 5b | Fig 6 | Supp Fig 1 |
| 1 | 1 | x |  | x | x (P) |  |  |  |  | x |
| 2 | 1 | x |  |  | x |  |  |  |  | x |
| 3 | 1 | x |  |  | x (P) |  |  |  |  | x |
| 4 | 1 | x |  |  | x (P) |  |  |  |  | x |
| 5 | 1 | x |  | x |  |  |  |  |  | x |
| 6 | 1 | x |  | x | x (P) | x | x | x |  | x |
| 7 | 2 | x |  | x |  |  |  |  |  | x |
| 8 | 2 | x |  |  | x (P) | x |  |  |  | x |
| 9 | 2 | x |  |  |  |  |  |  |  | x |
| 10 | 1 | x |  |  | x (P) |  |  |  |  | x |
| 11 | 1 | x (B) | x |  |  |  |  |  |  | x |
| 12 | 1 | x | x | x | x | x | x | x | x | x |
| 13 | 1 | x (B) | x | x | x | x |  |  |  | x |
| 14 | 1 | x | x | x | x |  |  |  |  | x |
| 15 | 1 | x (B) | x |  | x (P) | x |  | x |  | x |
| 16 | 2 | x (B) | x | x | x | x |  | x |  | x |
| 17 | 1 | x |  |  |  |  |  |  |  | x |
| 18 | 1 | x | x |  |  | x | x | x | x | x |
| 19 | 1 | x (B) | x |  | x | x | x | x | x | x |
| 20 | 1 | x | x | x | x | x | x | x |  | x |
| 21 | 2 | x | x | x | x | x | x | x | x | x |
| 22 | 1 | x | x | x | x |  |  |  | x | x |
| 23 | 1 | x | x | x |  |  |  |  |  | x |
| 24 | 1 | x | x | x | x | x |  | x | x | x |
| 25 | 2 | x | x | x | x | x | x | x | x | x |
| 26 | 2 | x |  | x | x | x | x | x | x | x |
| 27 | 2 | x |  | x |  |  |  |  |  | x |
| 28 | 1 | x | x | x | x | x |  | x | x | x |
| 29 | 2 | x | x | x | x |  |  |  | x | x |
| 30 | 1 | x | x | x | x | x | x | x | x | x |
| 31 | 2 | x | x | x | x | x | x | x | x | x |
| 32 | 2 | x | x | x | x | x |  | x | x | x |
| 33 | 2 | x | x | x | x | x |  | x | x | x |
| 34 | 2 | x | x | x | x | x | x | x | x | x |
| 35 | 2 | x | x | x | x | x |  |  | x | x |
| 36 | 2 | x | x | x | x | x | x | x | x | x |
| 37 | 2 | x | x | x | x | x | x | x | x | x |
| 38 | 2 | x |  |  |  |  |  |  |  |  |
| 39 | 1 | x | x | x | x |  | x | x |  | x |
| 40 | 2 | x | x | x | x | x | x | x | x | x |
| 41 | 2 | x | x | x |  | x |  | x |  |  |
| 42 | 2 | x | x | x | x | x |  | x | x | x |
| 43 | 2 | x | x | x | x | x | x | x | x | x |
| 44 | 1 | x | x | x |  |  |  |  |  | x |
| 45 | 2 | x | x | x |  |  |  |  |  | x |
| 46 | 2 | x |  |  |  |  |  |  |  |  |
| 47 | 1 | x | x | x |  |  |  |  |  | x |
| 48 | 2 | x | x | x |  |  |  |  |  | x |
| 49 | 1 | x | x | x |  |  |  |  |  | x |
| 50 | 1 | x | x |  | x | x | x | x | x | x |
| nClu1 | 26 | 26 | 18 | 16 | 18 | 11 | 8 | 10 | 8 | 26 |
| nClu2 | 24 | 24 | 17 | 20 | 16 | 16 | 9 | 14 | 14 | 21 |

**Supplementary Table 2: Electrophysiological properties of FS interneurons**

| Mean ± SEM | FS interneurons (n=3) |
| --- | --- |
| Resting membrane potential (mV) | -50 ± 4 |
| Membrane resistance (MΩ) | 465 ± 206 |
| Membrane time constant (ms) | 18 ± 8 |
| Sag Index (%) | 27 ± 4 |
| Rebound Index (%) | 46 ± 58 |
| Rheobase (pA) | 35 ± 27 |
| Delay to first spike (ms) | 31 ± 11 |
| AP threshold (mV) | -35 ± 10 |
| AP amplitude (mV) | 39.1 ± 2.5 |
| AP duration at half-width (ms) | 0.43 ± 0.12 |
| AP rise time (ms) | 0.58 ± 0.08 |
| AP rise slope (mV.ms^-1^) | 68.7 ± 6.4 |
| AP decay time (ms) | 0.33 ± 0.13 |
| AP decay slope (mV.ms^-1^) | 151 ± 40 |
| AP rise/decay slope ratio | 0.52 ± 0.13 |
| AHP amplitude (mV) | 26.1 ± 3.8 |
| fAHP amplitude (mV) | 26.1 ± 3.8 |
| ADP amplitude (mV) | 0 |
| mAHP amplitude (mV) | 0 |
| AHP duration (ms) | 0.92 ± 0.37 |
| ISI 1-2 (ms) | 34 ± 11 |
| Firing rate at +40 pA from rheobase (Hz) | 32 ± 16 |
| Early spike frequency adaptation | 1.06 ± 0.06 |
| Late spike frequency adaptation | 2.2 ± 1.0 |
| I/O gain (Hz.pA^-1^) | 1.72 ± 0.28 |
| Spontaneous frequency (Hz) | 0.044 ± 0.44 |
